# The interdisciplinary management of craniopharyngioma – practice patterns, outcomes, and insights

**DOI:** 10.1186/s12885-025-14991-3

**Published:** 2025-11-28

**Authors:** Jonas Haselmann, Siyer Roohani, David Wasilewski, Julia Onken, David Capper, David Kaul, Felix Ehret

**Affiliations:** 1 Charité – Universitätsmedizin Berlin, Corporate Member of Freie Universität Berlin and Humboldt-Universität zu Berlin, Department of Radiation Oncology, Berlin, Germany; 2 German Cancer Consortium (DKTK), partner site Berlin, a partnership between DKFZ and Charité – Universitätsmedizin Berlin, Berlin, Germany; 3 Charité – Universitätsmedizin Berlin, Corporate Member of Freie Universität Berlin and Humboldt-Universität zu Berlin, Department of Neurosurgery, Berlin, Germany; 4 Charité – Universitätsmedizin Berlin, Corporate Member of Freie Universität Berlin and Humboldt-Universität zu Berlin, Department of Neuropathology, Berlin, Germany; 5 Health and Medical University Potsdam, Potsdam, Germany

**Keywords:** Craniopharyngioma, Pediatrics, Neurosurgery, Radiotherapy, Radiation therapy, Stereotactic radiosurgery, Reirradiation, Quality of life, Hypothalamus, Pituitary gland, Neurocognitive deficits

## Abstract

**Background:**

Craniopharyngiomas are rare, mostly benign brain tumors, and their management remains challenging due to the limited data from large cohorts. This study evaluates the practice patterns and outcomes for craniopharyngioma patients managed at a tertiary care center.

**Methods:**

This retrospective cohort study included patients with histologically confirmed craniopharyngioma treated between 1996 and 2022. Patient, tumor, and treatment variables were analyzed for their association with local control (LC), progression-free survival (PFS), and overall survival (OS) using multivariable Cox regression models.

**Results:**

A total of 88 patients were analyzed. The median clinical and radiographic follow-up periods were 62.0 and 42.5 months, respectively. Fifty-three recurrences and twelve deaths were observed. After primary treatment, the 2-, 4, and 6-year LC and PFS rates were 69.1, 50.7, 37.7% and 71.5, 55.4, and 47.3%, respectively. For patients undergoing primary treatment, multivariable Cox regression showed an association between the extent of resection, i.e., gross total resection (GTR), and PFS (hazard ratio (HR): 0.36, *p* = 0.01) with weaker evidence for LC (HR: 0.40, *p* = 0.053). Age was the only variable associated with OS (HR: 1.05, *p* = 0.01). Seventeen patients received radiotherapy, which was not formally associated with LC, PFS, and OS. The majority of patients required hormone replacement therapy after treatment.

**Conclusions:**

This study underlines the role of GTR in delaying disease progression and the need for hormone replacement after treatment. While radiotherapy was not formally associated with any benefit in this series, its use might be helpful in candidates after subtotal resection and for treating recurrences. Further prospective research is needed to refine treatment algorithms, improve long-term outcomes, and optimize the quality of life of affected patients.

**Supplementary Information:**

The online version contains supplementary material available at 10.1186/s12885-025-14991-3.

## Background

Craniopharyngiomas are rare embryogenic tumors typically located along the pituitary–hypothalamic axis [1–3]. These tumors are generally considered benign and slow-growing [3]. Histopathologically, there are two subtypes, adamantinomatous and papillary craniopharyngioma [2]. Previous theories have postulated that the papillary subtype originates from the metaplasia of squamous epithelial cell remnants located in the region of the stomodeum that develops into the buccal mucosa [3]. The adamantinomatous type arises from epithelial remnants of the craniopharyngeal duct or Rathke’s pouch [3]. Craniopharyngiomas primarily occur in childhood and adolescence, as well as over the age of 40 [4, 5]. In childhood and adolescence, the predominant histology is adamantinomatous with cyst formation [6]. The papillary subtype is seen almost exclusively in adults, with an average patient age of 40–55 years [6, 7]. About 30–50% of all cases represent childhood craniopharyngioma [8, 9]. With an incidence of 0.5–2.5 new cases per 1 million population annually, it is a rare tumor entity [4, 8–10]. Most patients at diagnosis have neurological or endocrine dysfunctions, depending on the initial tumor volume and location at presentation [3]. Common symptoms in children and adults are headaches, visual impairment, nausea, and vomiting [11]. While children can suffer from growth failure, adults may suffer from hypogonadism and its sequelae [11]. Surgery and radiotherapy represent the central treatment modalities for craniopharyngioma and are used alone or in combination depending on the tumor location, tumor extension, symptoms, and previous treatments [11].

Given the rarity of the tumor, large contemporary studies with outcomes after interdisciplinary management are limited. Therefore, the purpose of this study is to report the treatment results of craniopharyngioma patients from a large tertiary center and to investigate the roles of the extent of resection and radiotherapy in the primary and recurrent setting.

## Materials and methods

All patients with a histopathologically confirmed diagnosis of a craniopharyngioma who were treated at our institution between 1996 and 2022 with at least one available follow-up were screened and included in this analysis. Two treatment periods were defined. The first period covers the outcomes in the primary treatment setting, i.e., the start of the first treatment until the diagnosis of the recurrence or death. The second period includes the time after the first recurrence until further disease progression or death. Patients were analyzed depending on data availability for the first and second periods. Endpoints of interest were local control (LC), progression-free survival (PFS), and overall survival (OS). LC was defined as the time from the start of therapy, i.e., surgery or definitive radiotherapy, to local recurrence or last radiographic follow-up in the absence of tumor progression. PFS was defined as the period from the start of therapy, i.e., surgery or definitive radiotherapy, to the first local recurrence or death of any cause. Local recurrence had to be confirmed by imaging with magnetic resonance imaging, with or without clinical deterioration, as assessed by the managing physicians. OS was defined as the time from initial resection to death of any cause. Patients without events were censored on the last available clinical follow-up for PFS and OS. The clinical follow-up was defined as the period between the first treatment and the last clinical contact. The radiographic follow-up was the period from the start of the first treatment to the last available imaging. For all analyses after the first recurrence, the diagnosis of local recurrence was the start for LC, PFS, OS, and follow-up calculations. If the exact day of treatment or recurrence was unknown, the 15th of the respective month was used. Cases with missing information on the day and month were excluded from the analysis. The Kaplan-Meier estimator has been used for the time-to-event analyses. Multivariable Cox regression analyses were performed to assess the association between patient, treatment, and tumor characteristics and outcomes of interest. Variable selection was done a priori and based on the available literature and previously reported findings [11]. The proportional hazards assumption was verified using a global test based on the Schoenfeld residuals and log-log plots. The goodness of fit of the models was assessed with the concordance index. A p-value of ≤ 0.05 was considered statistically significant. Percentages in this study are presented without rounding. The statistical analyses were done with SPSS Statistics v.29.0.1.1 (IBM Corp, Armonk, NY, USA) and STATA MP 17.0 (StataCorp, College Station, TX, USA). The study was approved by the local institutional review board (EA4/116/22) and follows the STROBE guidelines [12].

## Results

**Fig. 1:**
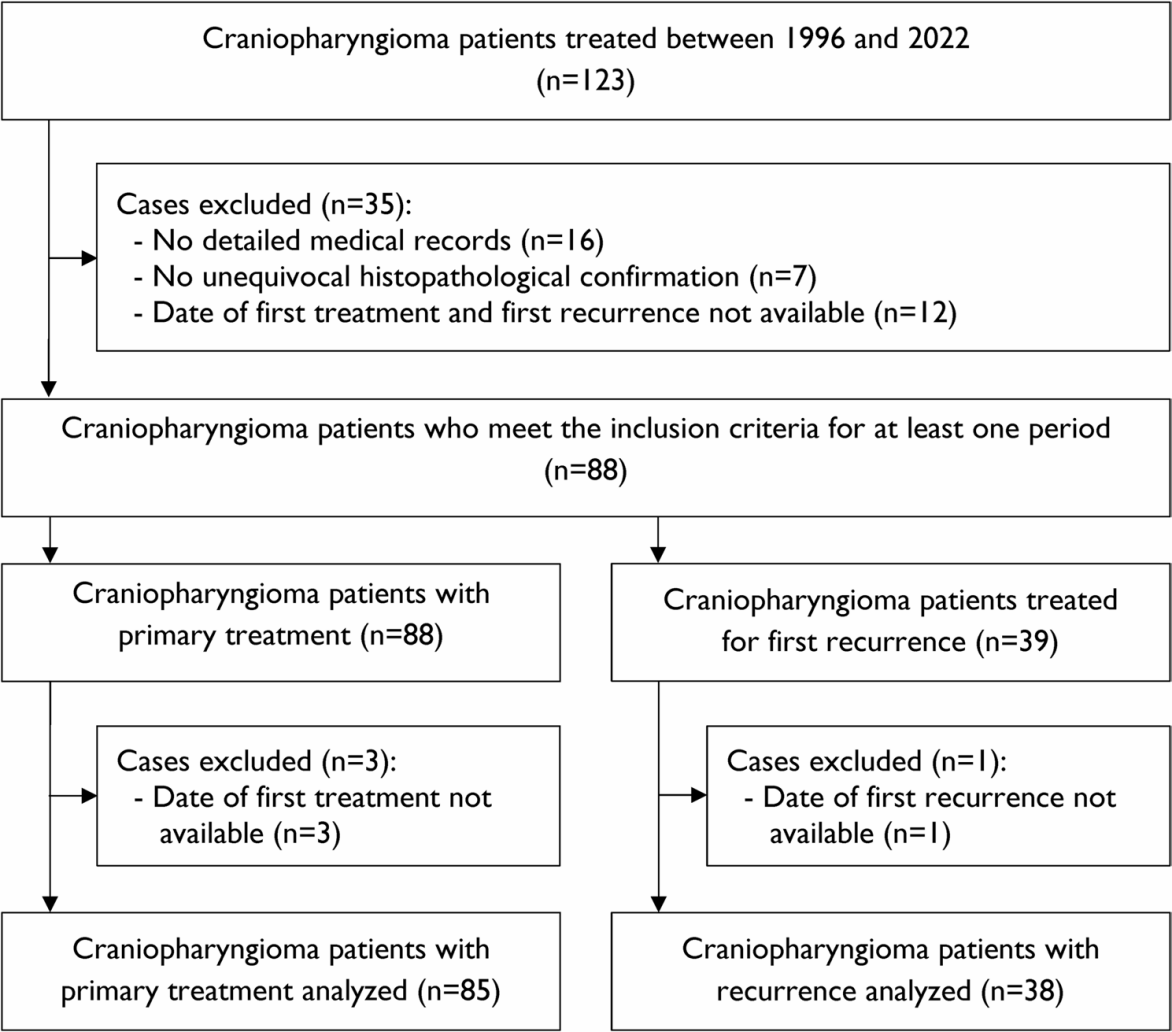
Flowchart of included and excluded patients

Between 1996 and 2022, 88 patients with a craniopharyngioma treated at our institution met the inclusion and exclusion criteria. The cohort flowchart is shown in Fig. 1.

### Primary treatment

#### Patient, tumor, and treatment characteristics

A total of 85 patients were analyzed for the primary disease setting. Over 50% of cases were treated between 2014 and 2021. Fifty-six patients (65.8%) had an adamantinomatous subtype, and 18 patients (21.1%) had a papillary subtype. The majority of patients was male (58.9%). The median age at primary therapy was 36.5 years (range 1.1–75.2). The most common symptoms at presentation were visual impairments (60.0%) and headaches (22.3%). All of the 85 patients underwent surgical resection, 33 of them (38.8%) had gross total resection (GTR), and 44 (51.7%) had subtotal resection (STR). In the remaining cases, the extent of resection was unavailable. A transcranial resection was performed in 65 patients (76.4%). Most patients had to be substituted with cortisol (88.2%), thyroid hormones (56.4%), and desmopressin (54.1%) after their surgery. One patient received only a biopsy and cyst drainage, which was counted as STR. Nine of the 85 patients (10.5%) underwent radiotherapy. One of the nine irradiated patients (1.1%) received definitive radiotherapy. The median prescription dose was 54 Gy (range 12–59.4), delivered in a median of 30 fractions (range 1–34). Three patients (3.5%) received intracystic interferon, and one patient (1.1%) received intracystic bleomycin. The patient, tumor, and treatment characteristics are summarized in Table 1.

**Table 1:** Patients, tumor, and treatment characteristics at first treatment

Number of patients	85													
Sex (number of male/female)	50 (58.9%)/35 (41.1%)													
Number of local recurrences/deaths	37 (43.5%)/12 (14.1%)													
Histology^a^	Adamantinomatous							Papillary						
	56 (65.8%)							18 (21.1%)						
Symptoms before first treatment^b^	Headache		Vomiting		Hormonal disorders	Vision impairment		Fatigue		Seizure		Cognitive disturbance		Neurological deficits
	19 (22.3%)		5 (5.8%)		8 (9.4%)	51 (60.0%)		7 (8.2%)		6 (7.0%)		11 (12.9%)		8 (9.4%)
Primary treatments	Surgery^c^							Radiotherapy						
	85 (100%)							9 (10.5%)						
	GTR^c^							STR^c, d^						
	33 (38.8%)							44 (51.7%)						
	TCR^e^							TSR^e^						
	65 (76.4%)							14 (16.4%)						
	Definitive radiotherapy^d^							Adjuvant radiotherapy						
	1 (1.1%)							8 (9.4%)						
Other treatments^f^	Intracystic interferon							Intracystic bleomycin						
	3 (3.5%)							1 (1.1%)						
Hormone replacement therapy after surgery^g^	Cortisol			Desmopressin			Thyroid hormones		Sex hormones				Growth hormones	
	75 (88.2%)			46 (54.1%)			48 (56.4%)		12 (14.1%)				2 (2.3%)	
Postoperative symptoms and neurocognitive impairment stratified by TCR/TSR^h^														
Developmental delay		5 (5.8%)/1 (1.1%)				Seizure					6 (7.0%)/0			
Headache		5 (5.8%)/4 (4.7%)				Vertigo and vomiting					2 (2.3%)/0			
Motor and sensory deficits		8 (9.4%)/1 (1.1%)				Depression					2 (2.3%)/0			
Impaired consciousness		5 (5.8%)/0				Fatigue					3 (3.5%)/0			
Addison crisis		2 (2.3%)/1 (1.1%)				Memory loss or attention deficit					3 (3.5%)/0			
Circadian rhythm disorder		2 (2.3%)/0				Delirium					6 (7.0%)/0			
Vision impairment		8 (9.4%)/0				Aggressive behavior					1 (1.1%)/0			
Cognitive impairment		2 (2.3%)/0				Adjustment disorder					1 (1.1%)/1 (1.1%)			
	Median				Mean (SD)			IQR				Range		
Age at primary treatment^i, j^	36.5				36.1 (23.0)			17.4–53.8				1.1–75.2		
Prescription dose (Gy)	54				49.6 (14.3)			51–54				12^k^−59.4		
Number of fractions	30				-			30–33				1–34		
Time from surgery to radiotherapy (months)^i^	2.3				2.9 (2.0)			1.2–4				0.8–7.2		
Clinical follow-up (months)^i^	62.0				79.1 (65.1)			29.4–119.1				0.2–286.7		
Radiographic follow-up (months)^i^	42.5				58.8 (59.6)			12.1–84.0				0.03–285.8		

*Abbreviations: GTR* Gross total resection, *STR* Subtotal resection, *TCR* Transcranial resection, *TSR* Transsphenoidal resection, *IQR* Interquartile range, *SD* Standard deviation, *Gy* Gray

^a^Subtype not available for eleven patients

^b^Data not available for 13 patients

^c^Extent of resection not available for eight patients

^d^One patient received a cyst drainage with biopsy, which was classified as STR. He underwent definitive radiotherapy

^e^Data not available for six patients

^f^Data not available for one patient

^g^Data not available for eight patients

^h^Data not available for nine patients

^i^When the exact day was not available, the 15th of the respective month was used for calculation. This was the case in five patients where only the month of the surgery was known

^j^Twenty-three of these patients were under 18 years old

^k^One patient received stereotactic radiosurgery

## Results

**Fig. 2:**
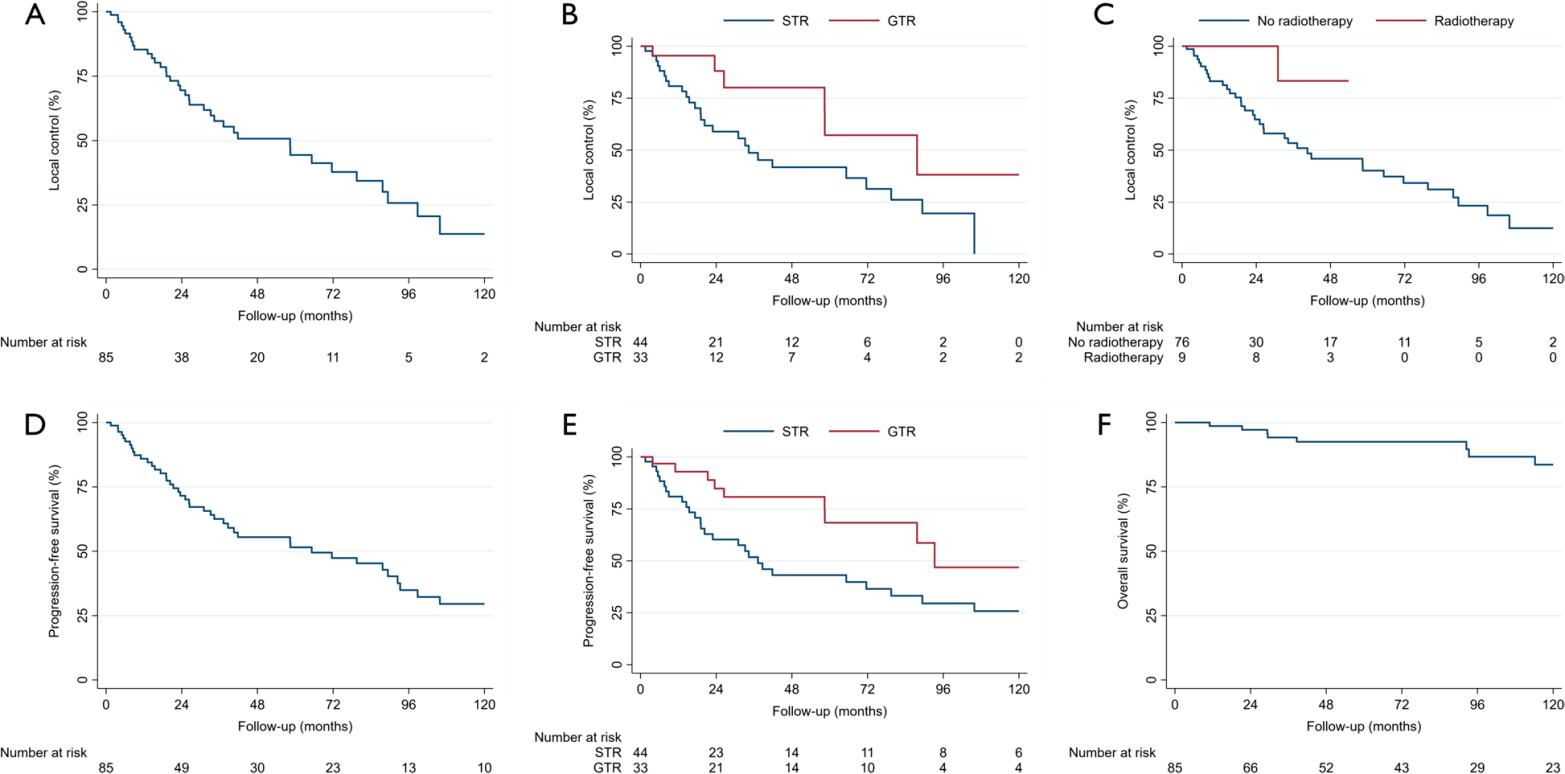
(**A**) Overall local control after primary treatment, (**B**) local control stratified for extent of resection after primary treatment, (**C**) local control stratified for radiotherapy or no radiotherapy after primary treatment, (**D**) overall progression-free survival after primary treatment, (**E**) progression-free survival stratified for extent of resection after primary treatment, (**F**) overall survival after primary treatment

The median clinical follow-up was 62.0 months (range 0.2–286.7), and the median radiographic follow-up was 42.5 months (range 0.03–285.8). During the available follow-up, 37 local recurrences (43.5%) and twelve deaths (14.1%) were observed (Table 1). The median LC was 58.3 months (95% confidence interval 31.0–79.5) (Fig. 2A). The 2-, 4, and 6-year LC rates were 69.1, 50.7, and 37.7%, respectively. Patients who underwent GTR had better LC rates compared to those with STR (Fig. 2B). Similarly, patients who received radiotherapy had prolonged LC compared to those who underwent surgery alone (Fig. 2C). All irradiated patients had undergone STR. Only one of these patients experienced a recurrence. However, neither radiotherapy nor the extent of resection was formally associated with LC (Table 2). The median PFS was 65.2 months (95% confidence interval 37.2–93.2). The 2-, 4, and 6-year PFS rates were 71.5, 55.4, and 47.3%, respectively. Regarding PFS, GTR showed an advantage over STR. Likewise, the PFS of irradiated patients was improved compared to non-irradiated patients. Histology did not influence PFS in this cohort. The PFS and OS for the entire cohort are shown in Fig. 2D and F. Figure 2E shows the PFS stratified by the extent of resection. A comparison of the outcomes between pediatric and adult patients and surgical techniques (transcranial vs. transsphenoidal) is provided in the Supplementary Materials 1 to 4. The most common postoperative complications were headaches (10.5%) and motor or sensory deficits (10.5%). Furthermore, 9.4% of patients experienced a deterioration in visual function compared with the preoperative status (Table 1, Supplementary Material 3). The multivariable Cox regression revealed a better PFS for patients undergoing GTR (HR: 0.36; *p* = 0.01). Although not statistically significant, a similar result was observed for LC (HR: 0.40; *p* = 0.053). Beyond that, patients without radiotherapy had a higher rate of local tumor progression (HR: 0.26, *p* = 0.20), although the observed evidence was weak. Age was the only variable associated with OS (HR: 1.05, *p* = 0.01). The multivariable Cox regressions did not identify other significantly associated variables for LC, PFS, and OS. The concordance indices of the multivariable Cox regressions for LC, PFS, and OS were 0.68, 0.69, and 0.86, respectively. The proportional hazards assumption was met for all analyses. The results of Cox regressions are summarized in Table 2.

**Table 2:** Multivariable cox regression analyses for local control, progression-free survival, and overall survival after primary treatment

Variable	Hazard ratio	Confidence interval (95%)	*p*-value
***Local control***			
Resection status			0.053
Subtotal resection	Reference		
Gross total resection	0.40	0.16–1.01	
Radiotherapy			0.13
No	Reference		
Yes	0.21	0.02–1.64	
Histology			0.39
Papillary	Reference		
Adamantinomatous	1.49	0.60–3.70	
***Progression-free survival***			
Resection status			0.01
Subtotal resection	Reference		
Gross total resection	0.36	0.15–0.81	
Radiotherapy			0.20
No	Reference		
Yes	0.26	0.03–2.08	
Histology			0.86
Papillary	Reference		
Adamantinomatous	1.07	0.46–2.50	
Age (in years)			0.30
	0.99	0.97–1.00	
***Overall survival***			
Resection status			0.52
Subtotal resection	Reference		
Gross total resection	1.75	0.30–10.17	
Radiotherapy			0.50
No	Reference		
Yes	2.26	0.20–24.89	
Histology			0.61
Papillary	Reference		
Adamantinomatous	0.64	0.11–3.53	
Age (in years)			0.01
	1.05	1.01–1.10	

### Outcomes after the first recurrence

#### Patient, tumor, and treatment characteristics

In total, 38 patients were analyzed for the second analysis period, while 35 of them were also part of the first period. More than 50% of cases were treated between 2017 and 2022. Most patients were male (60.5%). The adamantinomatous subtype was more common (76.3%). Only six patients (15.7%) had a papillary subtype. The median age at diagnosis of recurrence was 23.4 years (range 1.8–76.2). While 33 patients (86.8%) underwent surgery, eleven of them (28.9%) had a GTR, and 21 of them (55.2%) had a STR. The degree of resection for one patient was unknown. A transcranial resection was performed in 31 patients (81.5%). After the second surgery, several patients had to be newly substituted with desmopressin (18.4%), thyroid hormones (31.5%), and sex hormones (13.1%) for the first time. Eight patients (21.0%) underwent radiotherapy. Five of them received definitive radiotherapy, and the other three patients underwent adjuvant radiotherapy. The eight irradiated patients received a median of 16 fractions (range 1–32) with a median prescription dose of 33.1 Gy (range 12–54). Two patients (5.2%) received intracystic interferon, and one patient (2.6%) received intracystic bleomycin. The patient, tumor, and treatment characteristics for the recurrence cohort are summarized in Table 3.

**Table 3:** Patients, tumor, and treatment characteristics at first recurrence

Number of patients	38										
Number of patients already included in the first analysis period	35 (92.1%)										
Sex (number of male/female)	23 (60.5%)/15 (39.5%)										
Number of local recurrences/deaths	16 (42.1%)/4 (10.5%)										
Histology^a^	Adamantinomatous						Papillary				
	29 (76.3%)						6 (15.7%)				
Treatments for recurrence	Surgery^b^						Radiotherapy				
	33 (86.8%)						8 (21.0%)				
	GTR^b^						STR^b^				
	11 (28.9%)						21 (55.2%)				
	TCR						TSR				
	31 (81.5%)						2 (5.2%)				
	Definitive radiotherapy						Adjuvant radiotherapy				
	5 (13.1%)						3 (7.8%)				
New hormone replacement therapy after second surgery^c^	Cortisol		Desmopressin			Thyroid hormones		Sex hormones			Growth hormones
	1 (2.6%)		7 (18.4%)			12 (31.5%)		5 (13.1%)			3 (7.8%)
Postoperative symptoms and neurocognitive impairment stratified by TCR/TSR											
Developmental delay		7 (18.4%)/0			Seizure				3 (7.8%)/1 (2.6%)		
Headache		7 (18.4%)/0			Vertigo and vomiting				3 (7.8%)/0		
Motor and sensory deficits		2 (5.2%)/0			Depression				0/0		
Impaired consciousness		3 (7.8%)/0			Fatigue				2 (5.2%)/0		
Addison crisis		1 (2.6%)/0			Memory loss or attention deficit				2 (5.2%)/1 (2.6%)		
Circadian rhythm disorder		0/0			Delirium				1 (2.6%)/0		
Vision impairment		2 (5.2%)/0			Aggressive behavior				2 (5.2%)/0		
Cognitive impairment		0/0			Adjustment disorder				0/0		
Previous treatments	Surgery^d^						Radiotherapy				
	38 (100%)						1 (2.6%)				
	GTR^d^						STR^d^				
	6 (15.7%)						25 (65.7%)				
Other previous treatments	Intracystic interferon						Intracystic bleomycin				
	2 (5.2%)						1 (2.6%)				
	Median			Mean (SD)			IQR			Range	
Age at diagnosis of recurrence^e, f^	23.4			28.3 (21.1)			10.6–43.1			1.8–76.2	
Prescription dose (Gy)	33.1			33.3 (21.3)			13.2–54			12^g^ − 54	
Number of fractions	16			-			1–30			1–32	
Time from diagnosis to radiotherapy (months)^e^	8.3			9.7 (7.3)			4.5–13.4			1.1–23.9	
Clinical follow-up (months)^e^	53.0			65.6 (50.7)			33.5–88.7			2.4–213.5	
Radiographic follow-up (months)^e^	42.8			55.7 (48.5)			16.2–78.1			2.4–208.4	

*Abbreviations: GTR* Gross total resection, *STR* Subtotal resection, *TCR* Transcranial resection, *TSR* Transsphenoidal resection, *IQR* Interquartile range, *SD* Standard deviation, *Gy* Gray

^a^Data not available for three patients

^b^Data not available for one patient

^c^Data not available for two patients

^d^Extent of resection not available for seven patients

^e^When the exact day was not available, the 15th of the respective month was used for calculation. This was the case in three patients where only the month of the diagnosis of recurrence was known

^f^Fifteen of these patients were under 18 years old

^g^Four patients received stereotactic radiosurgery

## Results

**Fig. 3:**
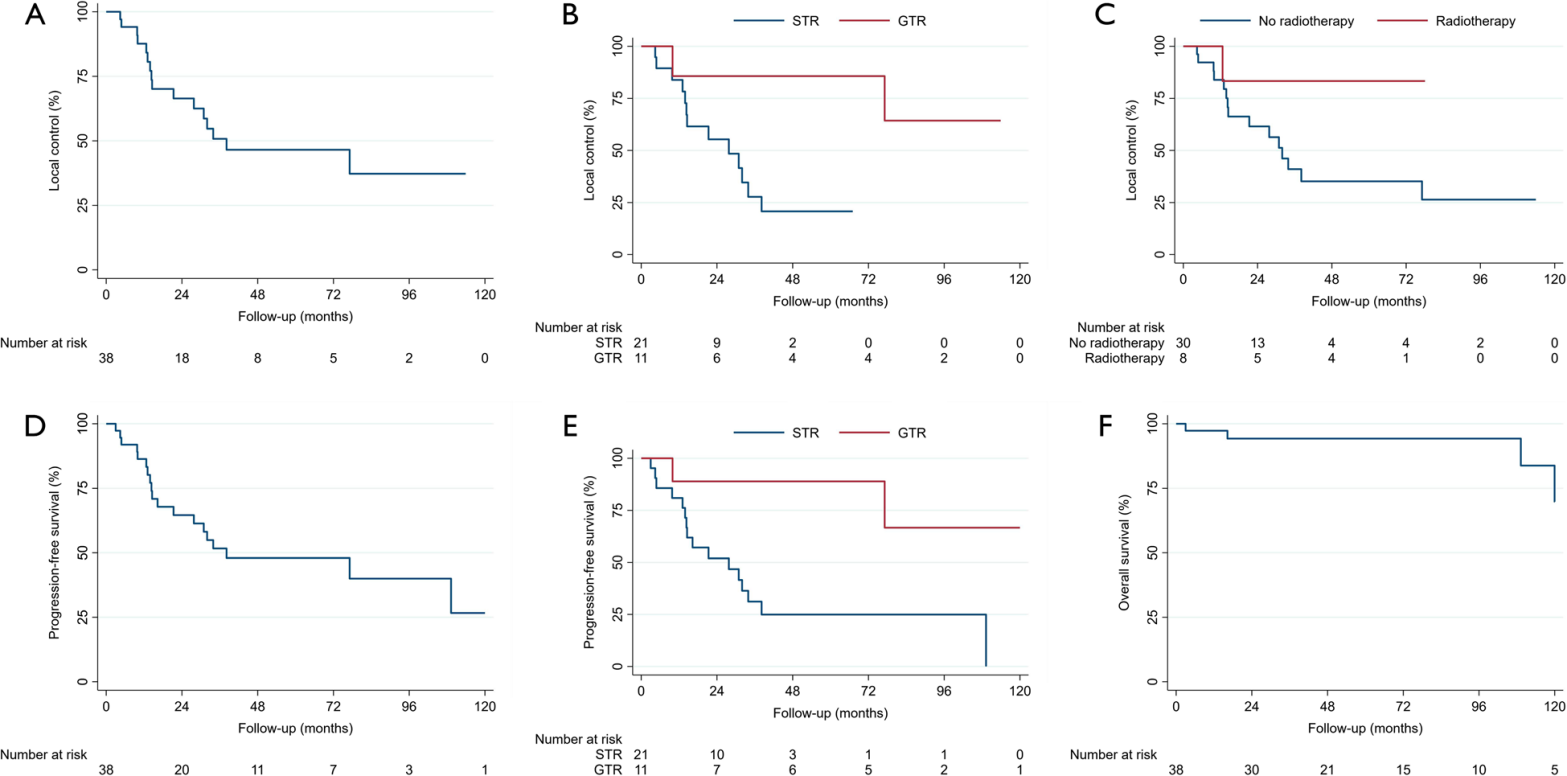
(**A**) Overall local control after first recurrence, (**B**) local control stratified for extent of resection after first recurrence, (**C**) local control stratified for radiotherapy or no radiotherapy after first recurrence, (**D**) overall progression-free survival after first recurrence, (**E**) progression-free survival stratified for extent of resection after first recurrence, (**F**) overall survival after first recurrence

**Fig. 4:**
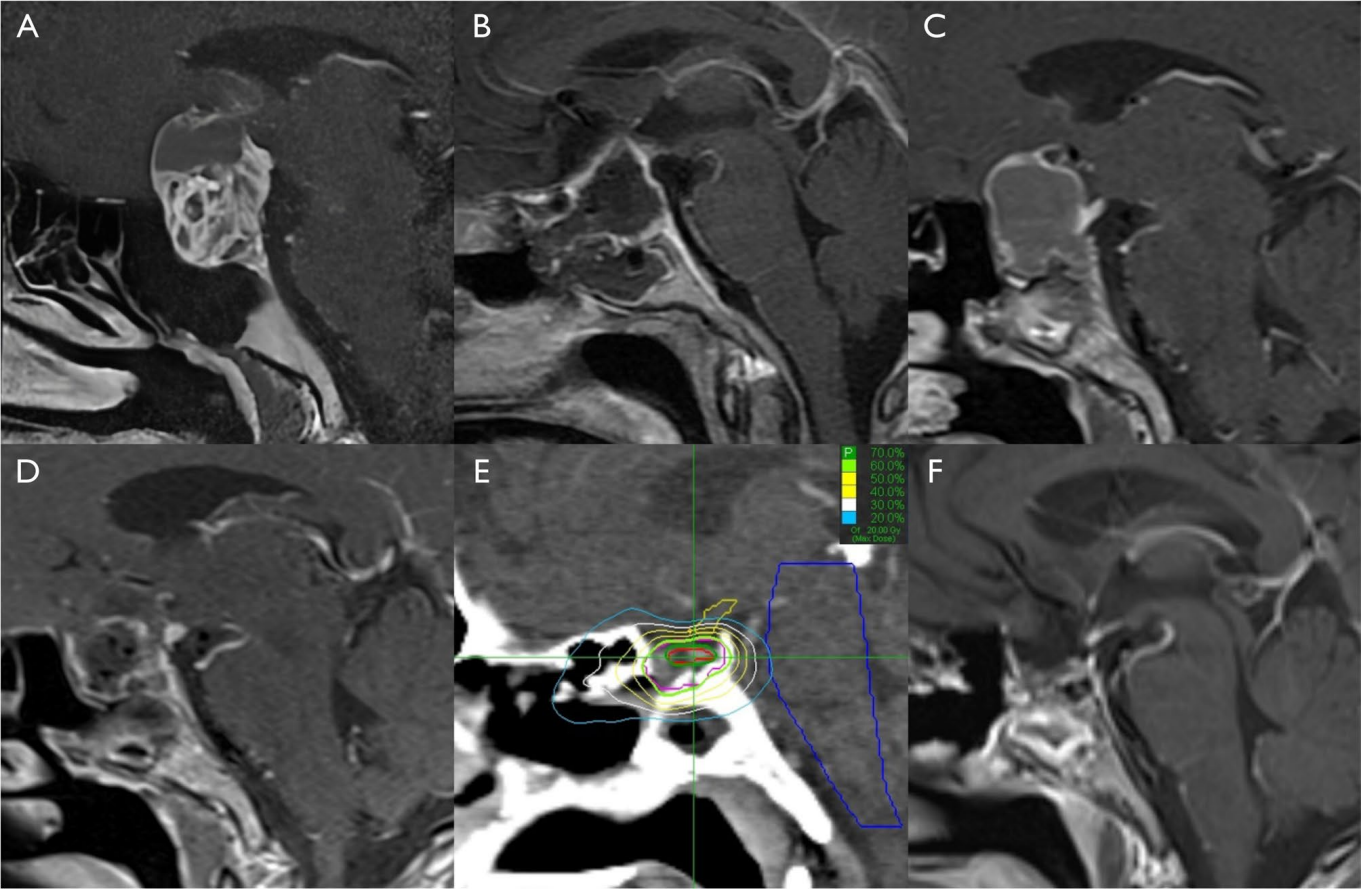
Case example. At the age of 26, the patient presented with visual impairment, specifically bitemporal hemianopsia. Subsequent magnetic resonance imaging (MRI) (**A**) revealed a 3 × 4 × 3 cm tumor. The tumor was surgically removed, and the histological diagnosis of an adamantinomatous craniopharyngioma was made. Follow-up imaging (**B**) revealed an irregularly shaped, rim-enhancing cystic formation, which, over time, was considered to be residual tumor tissue. Seven years after resection, the patient presented again with progressive visual deterioration. The following MRI (**C**) showed a three cm recurrence of the tumor, which was resected. Subsequent imaging (**D**) showed a residual cystic lesion with a contrast-enhancing capsule. Therefore, the decision for radiotherapy was made during an interdisciplinary tumor board meeting. Six months after the resection of the recurrence, the patient received stereotactic radiosurgery using a robotic radiosurgery system (CyberKnife) (**E**) with a dose of 12 Gy prescribed to the planning target volume and a dose of 14 Gy to the gross tumor volume. The prescription isodose line was 70%. Approximately two and a half years later, no progression of the residual tumor was observed in the most recent radiographic follow-up (**F**). At the time of the last clinical follow-up, the patient was suffering from anterior pituitary insufficiency, for which L-thyroxine, cortisol, and testosterone were being administered, as well as persistent visual impairment

The median clinical follow-up was 53.0 months (range 2.4–213.5), and the median radiographic follow-up was 42.8 months (range 2.4–208.4). During the available follow-up, 16 local recurrences (42.1%) and four deaths (10.5%) were observed (Table 3). The median LC was 38.1 months (95% confidence interval: 21.3 – not reached) (Fig. 3A). Patients who underwent GTR had better LC rates compared to those with STR (Fig. 3B). Similarly, patients who received radiotherapy had prolonged LC compared to those who underwent surgery alone (Fig. 3C). All irradiated patients had undergone STR. However, only one of these patients experienced a recurrence. The median PFS was 38.1 months (95% confidence interval: 16.2 – not reached). Regarding PFS, GTR showed an advantage over STR. Likewise, the PFS of irradiated patients was improved compared to non-irradiated patients. Histology also did not influence PFS in this cohort. The PFS and OS for the entire cohort are shown in Fig. 3D and F. Figure 3E shows the PFS stratified by the extent of resection. In addition to comparing outcomes between pediatric and adult patients and between surgical approaches, postoperative deficits in recurrent cases were also recorded, stratified by surgical approach (Supplementary Materials 1 to 4). In this cohort, the most common findings were headaches (18.4%) and developmental delay (18.4%) (Table 3, Supplementary Material 4). Due to the small sample size and low number of events, we did not proceed with multivariable Cox regression analyses for this cohort. An illustrative case is shown in Fig. 4.

## Discussion

Herein, we report a large contemporary series of craniopharyngioma patients treated at a large tertiary care center. In accordance with the available literature and previous reports, we observed an association between the extent of resection and outcomes [13–15]. While the degrees of evidence varied, and formal significance was only found for the association between resection status and PFS, our data highlight the central role of GTR in the successful management of craniopharyngioma patients [11]. Nevertheless, local tumor progression remains a central challenge. While the extent of resection decisively influences PFS, the OS of affected patients is excellent regardless of the surgical status. According to previous findings, an aggressive resection can lengthen the PFS but bears the risk of postoperative morbidity, which could influence the OS [16]. Therefore, the key to an optimal treatment is to balance local tumor control with treatment-associated toxicities. Surgery with or without radiotherapy or radiotherapy alone are the most commonly used treatment options [11]. Due to these nuances and the rarity of the tumor, each treatment has to be carefully tailored to the individual patient.

Various factors that influence the prognosis have been reported and discussed. For instance, a recent meta-analysis reports a higher risk of recurrence in adamantinomatous craniopharyngiomas [17]. Thirteen studies, including 974 patients, have been reviewed. Adamantinomatous craniopharyngiomas showed a total recurrence rate of 26.0% compared to the 14.1% of papillary tumors. Our multivariable Cox regression analysis did not detect a significant association between the histology and any endpoints of interest. The impact of histological subtypes remains controversial, and observed associations have not been reproduced in the available literature [11]. Other factors, such as hypothalamic involvement, neuroendocrine and neuropsychological deficits, and the number of surgical interventions, have also been reported to be of prognostic relevance. Still, the degrees of evidence are fairly limited, highlighting the existing controversies in the field [11].

Another treatment option to delay or prevent disease progression is radiotherapy. Various previous reports have assessed its role [18, 19]. Our results also suggest the benefit of radiotherapy to achieve durable tumor control. Although we lack strong evidence in our cohort due to the relatively small number of patients undergoing radiotherapy and the limited follow-up of these patients, radiotherapy remains particularly helpful in scenarios where safe GTR is not feasible. Ongoing discussions in the field include the role of proton therapy for craniopharyngioma patients, given its superior dosimetry over photon-based treatments. Due to the epidemiological challenges with this tumor entity, prospective studies are particularly limited. A recent phase 2 single-arm study investigated the outcomes after proton therapy in pediatric and adolescent craniopharyngioma patients [20]. A total of 94 patients were enrolled, and while the results of the co-primary endpoints, PFS and OS, did not markedly differ in comparison with a historical cohort, the neurocognitive outcomes were better after proton therapy. Sparing healthy brain tissue from radiation is of utmost importance in treating young craniopharyngioma patients. While access to proton therapy might be limited due to its high costs and the relatively low number of treatment facilities worldwide, stereotactic radiosurgery and fractionated stereotactic radiotherapy might be two alternatives for small tumors and local recurrences [18, 21]. Stereotactic radiosurgery was also used in four of our patients at first recurrence with favorable results.

In general, only a few studies address the detailed management of craniopharyngioma in the recurrent setting. Aside from this study, a recent systematic review and individual-participant meta-analysis from 2023 explored this topic, including eleven studies with a total of 80 patients out of 2932 reviewed studies [22]. Only patients who underwent surgical treatment for their primary disease were included in the study. The results consistently showed a benefit for patients receiving radiotherapy in the setting of local recurrence. Additionally, GTR was more effective than STR in delaying further tumor growth. The authors concluded that radiotherapy should be considered for patients with tumor progression following surgery alone. When radiotherapy cannot be administered, safe GTR should be prioritized as the treatment objective. The efficacy and safety of reirradiation in craniopharyngioma remain elusive [23].

Acute and late treatment-associated toxicity and morbidity play a central role in the management of craniopharyngioma. One of the most common difficulties is hormone deficiency following resection, which often requires hormone replacement therapy. Pituitary hormone deficiencies occur in 54–100% of patients [11, 24, 25]. Among them, adrenocorticotropic hormone deficiency is present in 55–88%, growth hormone deficiency in 88–100%, thyroid-stimulating hormone deficiency in 39–95%, and vasopressin deficiency in 25–86% of patients [11, 24, 25]. These percentages align well with our observations. The remaining discrepancies might be attributed to the percentages cited above being primarily based on pediatric studies and differences in tumor sizes and extent. In addition to hormonal disorders, resection of a craniopharyngioma is frequently associated with neurocognitive deficits, such as impairments in memory, attention, and cognitive processing speed [26]. Postoperative limitations in social and emotional functioning have also been reported in the literature and have a considerable impact on the patient’s quality of life [11]. We have also observed a variety of neurocognitive deficits after treatment, underlining the need to individualize care and limit tumor- and treatment-related deficits. A carefully planned surgery remains a key to achieving this objective. A direct comparison between transcranial and transsphenoidal approaches with respect to neurocognitive outcomes is largely lacking, although previous studies have examined the relationship between these surgical techniques and overall neurological complications [27].

Radiotherapy might also cause treatment-related toxicity. Common concerns after radiotherapy at a young age include the development of second malignancies. A multicenter cohort study analyzing 3679 patients with pituitary adenoma or craniopharyngioma found a cumulative probability of secondary malignancy of 4% after 20 years for the 996 patients receiving radiotherapy [28]. In contrast, the risk was 2.1% for patients not receiving radiation. Notably, more than 45% of irradiated patients received 2-dimensional radiotherapy, and only 4.3% underwent intensity-modulated radiotherapy. Therefore, it remains unclear whether the risk of secondary malignancies is comparable with modern radiation techniques such as volumetric modulated arc therapy, stereotactic radiosurgery, or proton therapy. All these tumor- and treatment-associated symptoms and adverse effects can lead to a considerable burden for affected patients. Various studies and reviews highlight the reduced quality of life for patients with craniopharyngiomas compared to the general population [29]. Despite the considerable risk of bias of the analyzed studies, a recent systematic review underlined the iatrogenic contribution to the poor quality of life, calling for further refinements in surgery and radiotherapy [29].

While both have been the treatment modalities of choice for decades, recent advances in molecular neuropathology and targeted therapies offer new avenues in managing craniopharyngiomas. Papillary craniopharyngiomas frequently exhibit BRAF V600E mutations [30]. Over 90% of tumors harbor such mutations, making them a preferred treatment target [30]. In a recent phase 2 study from 2023, 16 patients with papillary craniopharyngioma were treated with the BRAF-MEK inhibitor combination vemurafenib-cobimetinib [31]. Fifteen of those patients had a durable response to the treatment, with a median reduction of the tumor volume of 91% [31]. The patients’ PFS was 87% at 12 months and 58% at 24 months, highlighting the efficacy of the targeted therapy [31]. However, it has to be noted that three patients had to stop treatment due to adverse events. While BRAF mutations represent a target for papillary tumors, adamantinomatous craniopharyngiomas currently lack such a target.

This study has several limitations that must be acknowledged when interpreting the reported findings. While the sample size of analyzed patients is considerable, it is still insufficient to detect subtle treatment effects and other factors associated with the investigated outcomes, particularly after treatment for local tumor recurrence. This limitation is further pronounced given the lack of long-term follow-up in a subset of patients. Moreover, missing data on some of the investigated variables, including side effects and treatment-associated toxicity, represent another limitation, which also affected the statistical power of our multivariable Cox regression analyses. Finally, given that all patients have been treated at a tertiary care center, an underlying sampling bias of cases has to be anticipated.

## Conclusion

Craniopharyngiomas are rare tumors requiring interdisciplinary management, with individualized treatment approaches, depending on the patient’s age, tumor histology, size and extent, previous treatments, and symptoms. This study supports the role of surgical resection and radiotherapy in managing these tumors. Despite the favorable survival prognosis, many patients will experience local tumor progression, warranting safe and effective salvage treatment modalities. Future research needs to define the roles of advanced surgical and radiation techniques, the efficacy and safety of reirradiation, the timing of radiotherapy, and targeted therapies. Finally, it is critical to find new ways to improve the long-term quality of life of affected patients and limit treatment- and tumor-related symptoms.

## Supplementary Information

Supplementary Material 1. (A) Overall local control stratified for age after primary treatment, (B) progression-free survival stratified for age after primary treatment, (C) overall survival stratified for age after primary treatment, (D) overall local control stratified for transcranial or transsphenoidal resection after primary treatment, (E) progression-free survival stratified for transcranial or transsphenoidal resection after primary treatment, (F) overall survival for transcranial or transsphenoidal resection after primary treatment

Supplementary Material 2. (A) Overall local control stratified for age after first recurrence, (B) progression-free survival stratified for age after first recurrence, (C) overall survival stratified for age after first recurrence, (D) overall local control stratified for transcranial or transsphenoidal resection after first recurrence, (E) progression-free survival stratified for transcranial or transsphenoidal resection after first recurrence, (F) overall survival for transcranial or transsphenoidal resection after first recurrence

Supplementary Material 3. Patients, tumor, and treatment characteristics at first treatment stratified by age group

Supplementary Material 4. Patients, tumor, and treatment characteristics at first recurrence stratified by age group

## Data Availability

The data underlying the analysis can be obtained from the corresponding author upon reasonable request.

## Abbreviations

LC: local control
PFS: progression–free survival (PFS)
OS: overall survival
GTR: gross total resection
STR: subtotal resection
IQR: Interquartile range
SD: Standard deviation
Gy: Gray
