# Supplementary figures and images for: The interdisciplinary management of craniopharyngioma – practice patterns, outcomes, and insights
Source: BMC Cancer. 2025 Nov 28;25:1837. doi: 10.1186/s12885-025-14991-3 (PMC12667174; doi:10.1186/s12885-025-14991-3)

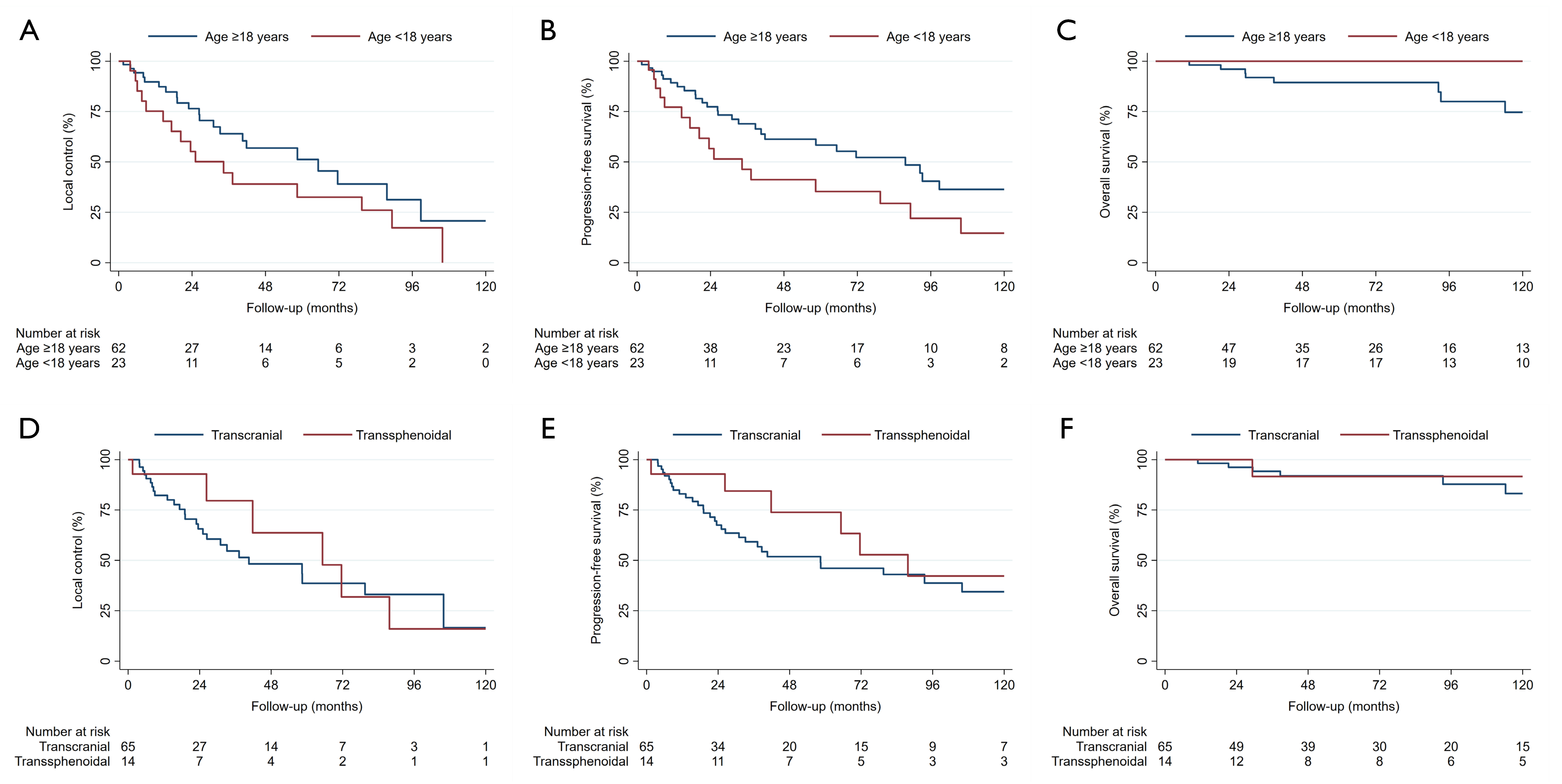

Supplement: Supplementary file 1 — Supplementary Material 1. (A) Overall local control stratified for age after primary treatment, (B) progression-free survival stratified for age after primary treatment, (C) overall survival stratified for age after primary treatment, (D) overall local control stratified for transcranial or transsphenoidal resection after primary treatment, (E) progression-free survival stratified for transcranial or transsphenoidal resection after primary treatment, (F) overall survival for transcranial or transsphenoidal resection after primary treatment [file 12885_2025_14991_MOESM1_ESM.jpg]

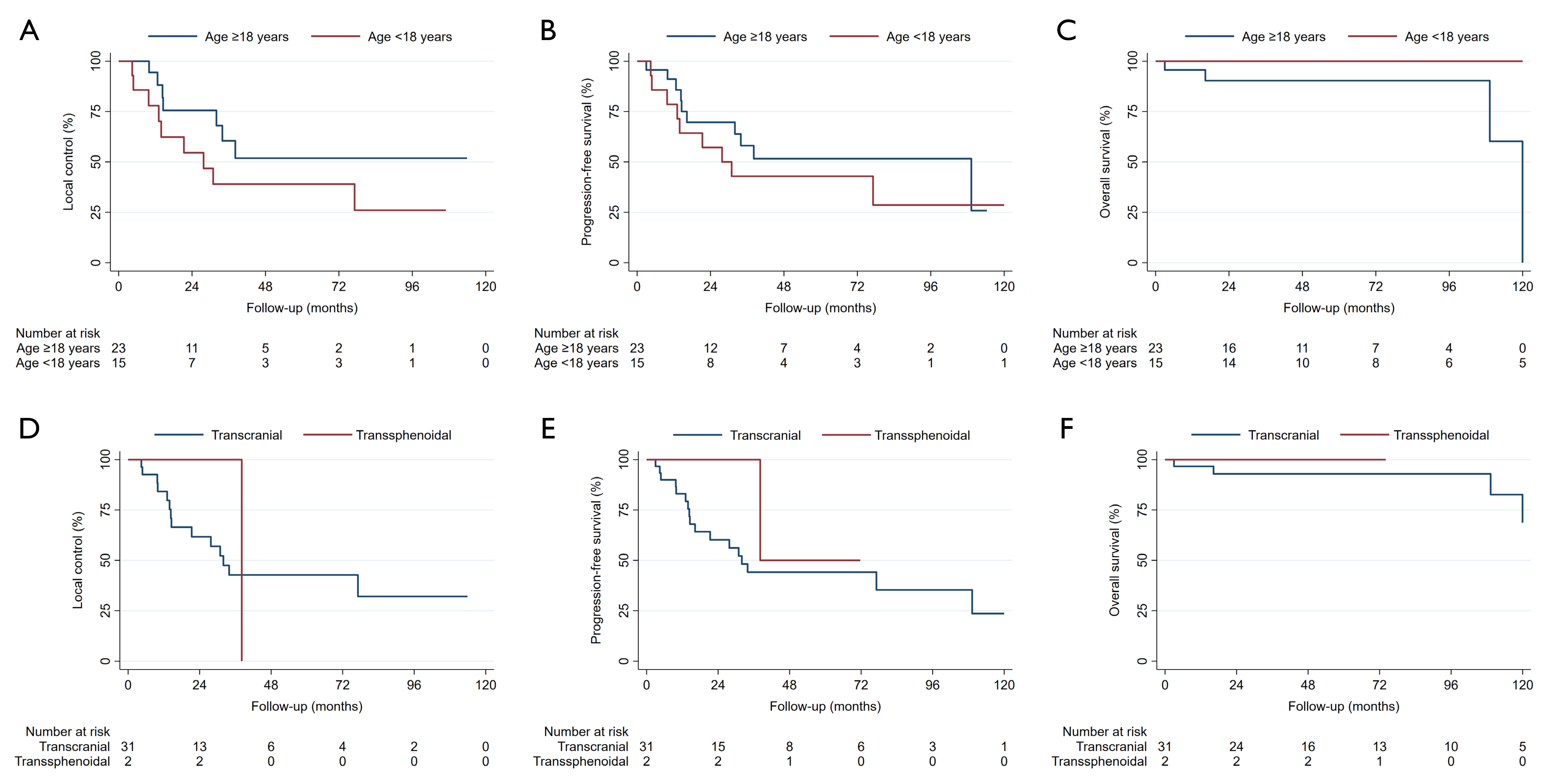

Supplement: Supplementary file 2 — Supplementary Material 2. (A) Overall local control stratified for age after first recurrence, (B) progression-free survival stratified for age after first recurrence, (C) overall survival stratified for age after first recurrence, (D) overall local control stratified for transcranial or transsphenoidal resection after first recurrence, (E) progression-free survival stratified for transcranial or transsphenoidal resection after first recurrence, (F) overall survival for transcranial or transsphenoidal resection after first recurrence [file 12885_2025_14991_MOESM2_ESM.jpg]
