# Supplementary material for: The interdisciplinary management of craniopharyngioma – practice patterns, outcomes, and insights
Source: BMC Cancer. 2025 Nov 28;25:1837. doi: 10.1186/s12885-025-14991-3 (PMC12667174; doi:10.1186/s12885-025-14991-3)
Supplement: Supplementary file 3 — Supplementary Material 3. Patients, tumor, and treatment characteristics at first treatment stratified by age group [file 12885_2025_14991_MOESM3_ESM.docx]

**Supplementary File 3.** Patients, tumor, and treatment characteristics at first treatment stratified by age group.

| **Number of patients under 18 years** | 23 (27.0%) | | | | | | | | | | | | | | |
| --- | --- | --- | --- | --- | --- | --- | --- | --- | --- | --- | --- | --- | --- | --- | --- |
| Sex (number of male/female) | 14 (60.8%)/9 (39.2%) | | | | | | | | | | | | | | |
| Number of local recurrences/deaths | 16 (69.5%)/0 | | | | | | | | | | | | | | |
| Histology^a^ | **Adamantinomatous** | | | | | | | | **Papillary** | | | | | | |
|  | 19 (82.6%) | | | | | | | | 2 (8.6%) | | | | | | |
| Symptoms before first treatment^b^ | **Headache** | | **Vomiting** | | **Hormonal disorders** | | **Vision impairment** | | **Fatigue** | | **Seizure** | | **Cognitive disturbance** | | **Neurological**  **deficits** |
|  | 8  (34.7%) | | 5  (21.7%) | | 3  (13.0%) | | 10  (43.4%) | | 2  (8.6%) | | 4  (17.3%) | | 4  (17.3%) | | 3  (13.0%) |
| Primary treatments | **Surgery**^c^ | | | | | | | | **Radiotherapy** | | | | | | |
|  | 23 (100%) | | | | | | | | 1 (4.3%) | | | | | | |
|  | **GTR**^c^ | | | | | | | | **STR**^c^ | | | | | | |
|  | 4 (17.3%) | | | | | | | | 17 (73.9%) | | | | | | |
|  | **TCR**^d^ | | | | | | | | **TSR**^d^ | | | | | | |
|  | 17 (73.9%) | | | | | | | | 2 (8.6%) | | | | | | |
|  | **Definitive radiotherapy**^e^ | | | | | | | | **Adjuvant radiotherapy** | | | | | | |
|  | 1 (4.3%) | | | | | | | | 0 | | | | | | |
| Other treatments | **Intracystic interferon** | | | | | | | | **Intracystic bleomycin** | | | | | | |
|  | 3 (13.0%) | | | | | | | | 1 (4.3%) | | | | | | |
| Hormone replacement therapy after surgery^f^ | **Cortisol** | | | **Desmopressin** | | | | **Thyroid hormones** | | **Sex hormones** | | | | **Growth hormones** | |
|  | 20 (86.9%) | | | 15 (65.2%) | | | | 11 (47.8%) | | 1 (4.3%) | | | | 1 (4.3%) | |
| **Postoperative symptoms and neurocognitive impairment stratified by TCR/TSR**^b^ | | | | | | | | | | | | | | | |
| **Developmental delay** | | 5 (21.7%)/1 (4.3%) | | | | **Seizure** | | | | | | 2 (8.6%)/0 | | | |
| **Headache** | | 1 (4.3%)/1 (4.3%) | | | | **Vertigo and vomiting** | | | | | | 0/0 | | | |
| **Motor and sensory deficits** | | 1 (4.3%)/0 | | | | **Depression** | | | | | | 1 (4.3%)/0 | | | |
| **Impaired consciousness** | | 0/0 | | | | **Fatigue** | | | | | | 0/0 | | | |
| **Addison crisis** | | 1 (4.3%)/0 | | | | **Memory loss or attention deficit** | | | | | | 2 (8.6%)/0 | | | |
| **Circadian rhythm disorder** | | 1 (4.3%)/0 | | | | **Delirium** | | | | | | 0/0 | | | |
| **Vision impairment** | | 1 (4.3%)/0 | | | | **Aggressive behavior** | | | | | | 0/0 | | | |
| **Cognitive impairment** | | 0/0 | | | | **Adjustment disorder** | | | | | | 1 (4.3%)/0 | | | |
| **Number of patients 18 years or older** | 62 (73.0%) | | | | | | | | | | | | | | |
| Sex (number of male/female) | 36 (58.0%)/26 (42.0%) | | | | | | | | | | | | | | |
| Number of local recurrences/deaths | 21 (33.8%)/12 (19.3%) | | | | | | | | | | | | | | |
| Histology^g^ | **Adamantinomatous** | | | | | | | | **Papillary** | | | | | | |
|  | 37 (59.6%) | | | | | | | | 16 (25.8%) | | | | | | |
| Symptoms before first treatment^h^ | **Headache** | | **Vomiting** | | **Hormonal disorders** | | **Vision impairment** | | **Fatigue** | | **Seizure** | | **Cognitive disturbance** | | **Neurological**  **deficits** |
|  | 11 (17.7%) | | 0 | | 5  (8.0%) | | 41  (66.1%) | | 5  (8.0%) | | 2  (3.2%) | | 7  (11.2%) | | 5  (8.0%) |
| Primary treatments | **Surgery**^i^ | | | | | | | | **Radiotherapy** | | | | | | |
|  | 62 (100%) | | | | | | | | 8 (12.9%) | | | | | | |
|  | **GTR**^i^ | | | | | | | | **STR**^i^ | | | | | | |
|  | 29 (46.7%) | | | | | | | | 27 (43.5%) | | | | | | |
|  | **TCR**^c^ | | | | | | | | **TSR**^c^ | | | | | | |
|  | 48 (77.4%) | | | | | | | | 12 (19.3%) | | | | | | |
|  | **Definitive radiotherapy** | | | | | | | | **Adjuvant radiotherapy** | | | | | | |
|  | 0 | | | | | | | | 8 (12.9%) | | | | | | |
| Other treatments^j^ | **Intracystic interferon** | | | | | | | | **Intracystic bleomycin** | | | | | | |
|  | 0 | | | | | | | | 0 | | | | | | |
| Hormone replacement therapy after surgery^b^ | **Cortisol** | | | **Desmopressin** | | | | **Thyroid hormones** | | **Sex hormones** | | | | **Growth hormones** | |
|  | 55 (88.7%) | | | 31 (50.0%) | | | | 37 (59.6%) | | 11 (17.7%) | | | | 1 (1.6%) | |
| **Postoperative symptoms and neurocognitive impairment stratified by TCR/TSR**^d^ | | | | | | | | | | | | | | | |
| **Developmental delay** | | 0/0 | | | | **Seizure** | | | | | | 4 (6.4%)/0 | | | |
| **Headache** | | 4 (6.4%)/3 (4.8%) | | | | **Vertigo and vomiting** | | | | | | 2 (3.2%)/0 | | | |
| **Motor and sensory deficits** | | 7 (11.2%)/1 (1.6%) | | | | **Depression** | | | | | | 1 (1.6%)/0 | | | |
| **Impaired consciousness** | | 5 (8.0%)/0 | | | | **Fatigue** | | | | | | 3 (4.8%)/0 | | | |
| **Addison crisis** | | 1 (1.6%)/1 (1.6%) | | | | **Memory loss or attention deficit** | | | | | | 1 (1.6%)/0 | | | |
| **Circadian rhythm disorder** | | 1 (1.6%)/0 | | | | **Delirium** | | | | | | 6 (9.6%)/0 | | | |
| **Vision impairment** | | 7 (11.2%)/0 | | | | **Aggressive behavior** | | | | | | 1 (1.6%)/0 | | | |
| **Cognitive impairment** | | 2 (3.2%)/0 | | | | **Adjustment disorder** | | | | | | 0/1 (1.6%) | | | |

Abbreviations: GTR = Gross total resection, STR = Subtotal resection, TCR = Transcranial resection, TSR = Transsphenoidal resection.
^a^Subtype not available for two patients.

^b^Data not available for five patients. ^c^Data not available for two patients.

^d^Data not available for four patients.

^e^One patient received a cyst drainage with biopsy, which was classified as STR. He underwent definitive radiotherapy.

^f^Data not available for three patients.

^g^Subtype not available for nine patients.

^h^Data not available for eight patients.

^i^Data not available for six patients.

^j^Data not available for one patient.
