# Supplementary material for: The interdisciplinary management of craniopharyngioma – practice patterns, outcomes, and insights
Source: BMC Cancer. 2025 Nov 28;25:1837. doi: 10.1186/s12885-025-14991-3 (PMC12667174; doi:10.1186/s12885-025-14991-3)
Supplement: Supplementary file 4 — Supplementary Material 4. Patients, tumor, and treatment characteristics at first recurrence stratified by age group [file 12885_2025_14991_MOESM4_ESM.docx]

**Supplementary File 4.** Patients, tumor, and treatment characteristics at first recurrence stratified by age group.

| **Number of patients under 18 years** | 15 (39.4%) | | | | | | | | | | |
| --- | --- | --- | --- | --- | --- | --- | --- | --- | --- | --- | --- |
| Number of patients already included in the first analysis period | 14 (93.3%) | | | | | | | | | | |
| Sex (number of male/female) | 10 (66.6%)/5 (33.4%) | | | | | | | | | | |
| Number of local recurrences/deaths | 9 (60%)/0 | | | | | | | | | | |
| Histology | **Adamantinomatous** | | | | | | | **Papillary** | | | |
|  | 14 (93.3%) | | | | | | | 1 (6.7%) | | | |
| Treatments for recurrence | **Surgery** | | | | | | | **Radiotherapy** | | | |
|  | 15 (100%) | | | | | | | 1 (6.6%) | | | |
|  | **GTR** | | | | | | | **STR** | | | |
|  | 5 (33.4%) | | | | | | | 10 (66.6%) | | | |
|  | **TCR** | | | | | | | **TSR** | | | |
|  | 15 (100%) | | | | | | | 0 | | | |
|  | **Definitive radiotherapy** | | | | | | | **Adjuvant radiotherapy** | | | |
|  | 0 | | | | | | | 1 (6.6%) | | | |
| New hormone replacement therapy after second surgery^a^ | **Cortisol** | | | **Desmopressin** | | **Thyroid hormones** | | | **Sex hormones** | | **Growth hormones** |
|  | 0 | | | 1 (6.6%) | | 5 (33.3%) | | | 1 (6.6%) | | 3 (20.0%) |
| **Postoperative symptoms and neurocognitive impairment stratified by TCR/TSR** | | | | | | | | | | | |
| **Developmental delay** | | 6 (40.0%)/0 | | | **Seizure** | | | | | 2 (13.3%)/0 | |
| **Headache** | | 3 (20.0%)/0 | | | **Vertigo and vomiting** | | | | | 2 (13.3%)/0 | |
| **Motor and sensory deficits** | | 1 (6.6%)/0 | | | **Depression** | | | | | 0/0 | |
| **Impaired consciousness** | | 1 (6.6%)/0 | | | **Fatigue** | | | | | 0/0 | |
| **Addison crisis** | | 1 (6.6%)/0 | | | **Memory loss or attention deficit** | | | | | 0/0 | |
| **Circadian rhythm disorder** | | 0/0 | | | **Delirium** | | | | | 0/0 | |
| **Vision impairment** | | 1 (6.6%)/0 | | | **Aggressive behavior** | | | | | 1 (6.6%)/0 | |
| **Cognitive impairment** | | 0/0 | | | **Adjustment disorder** | | | | | 0/0 | |
| Previous treatments | **Surgery** | | | | | | **Radiotherapy** | | | | |
|  | 15 (100%) | | | | | | 0 | | | | |
|  | **GTR** | | | | | | **STR** | | | | |
|  | 2 (13.3%) | | | | | | 13 (86.7%) | | | | |
| Other previous treatments | **Intracystic interferon** | | | | | | **Intracystic bleomycin** | | | | |
|  | 2 (13.3%) | | | | | | 1 (6.6%) | | | | |
| **Number of patients 18 years or older** | 23 (60.5%) | | | | | | | | | | |
| Number of patients already included in the first analysis period | 21 (91.3%) | | | | | | | | | | |
| Sex (number of male/female) | 13 (56.5%)/10 (43.5%) | | | | | | | | | | |
| Number of local recurrences/deaths | 7 (30.4%)/4 (17.3%) | | | | | | | | | | |
| Histology^b^ | **Adamantinomatous** | | | | | | | **Papillary** | | | |
|  | 15 (65.2%) | | | | | | | 5 (21.7%) | | | |
| Treatments for recurrence | **Surgery**^a^ | | | | | | | **Radiotherapy** | | | |
|  | 18 (78.2%) | | | | | | | 7 (30.4%) | | | |
|  | **GTR**^a^ | | | | | | | **STR**^a^ | | | |
|  | 6 (26.0%) | | | | | | | 11 (47.8%) | | | |
|  | **TCR** | | | | | | | **TSR** | | | |
|  | 16 (69.5%) | | | | | | | 2 (8.6%) | | | |
|  | **Definitive radiotherapy** | | | | | | | **Adjuvant radiotherapy** | | | |
|  | 5 | | | | | | | 2 | | | |
| New hormone replacement therapy after second surgery^a^ | **Cortisol** | | **Desmopressin** | | | **Thyroid hormones** | | | **Sex hormones** | | **Growth hormones** |
|  | 1 (4.3%) | | 6 (26.0%) | | | 7 (30.4%) | | | 4 (17.3%) | | 0 |
| **Postoperative symptoms and neurocognitive impairment stratified by TCR/TSR** | | | | | | | | | | | |
| **Developmental delay** | | 1 (4.3%)/0 | | | **Seizure** | | | | | 1 (4.3%)/1 (4.3%) | |
| **Headache** | | 4 (17.3%)/0 | | | **Vertigo and vomiting** | | | | | 1 (4.3%)/0 | |
| **Motor and sensory deficits** | | 1 (4.3%)/0 | | | **Depression** | | | | | 0/0 | |
| **Impaired consciousness** | | 2 (8.6%)/0 | | | **Fatigue** | | | | | 2 (8.6%)/0 | |
| **Addison crisis** | | 0/0 | | | **Memory loss or attention deficit** | | | | | 2 (8.6%)/1 (4.3%) | |
| **Circadian rhythm disorder** | | 0/0 | | | **Delirium** | | | | | 1 (4.3%)/0 | |
| **Vision impairment** | | 1 (4.3%)/0 | | | **Aggressive behavior** | | | | | 1 (4.3%)/0 | |
| **Cognitive impairment** | | 0/0 | | | **Adjustment disorder** | | | | | 0/0 | |
| Previous treatments | **Surgery**^c^ | | | | | | **Radiotherapy** | | | | |
|  | 23 (100%) | | | | | | 1 (4.3%) | | | | |
|  | **GTR**^c^ | | | | | | **STR**^c^ | | | | |
|  | 4 (17.3%) | | | | | | 12 (52.1%) | | | | |
| Other previous treatments^d^ | **Intracystic interferon** | | | | | | **Intracystic bleomycin** | | | | |
|  | 0 | | | | | | 0 | | | | |

Abbreviations: GTR = Gross total resection, STR = Subtotal resection, TCR = Transcranial resection, TSR = Transsphenoidal resection.
^a^Data not available for one patient.

^b^Data not available for three patients.

^c^Data not available for seven patients.

^d^Data not available for eight patients.
